# General Population and Healthcare Professionals' Level of Knowledge of the Burden and Prevention of Respiratory Syncytial Virus in France

**DOI:** 10.1111/irv.70103

**Published:** 2025-04-28

**Authors:** Joshua Puel, Katia Sosnowiez, Robin Stephan, Albert Sotto, Paul Loubet

**Affiliations:** ^1^ VBIC, INSERM U1047, Univ Montpellier Service des Maladies Infectieuses et Tropicales, CHU Nîmes Nîmes France; ^2^ Jannssen‐Cilag ‐ Issy‐Les‐Moulineaux Paris France; ^3^ VBIC, INSERM U1047, Univ Montpellier Service de Microbiologie et Hygiène Hospitalière, CHU Nîmes Nîmes France

**Keywords:** burden, knowledge, respiratory viruses, RSV, vaccines

## Abstract

**Introduction:**

This study aimed to assess the level of knowledge among the general population and French healthcare professionals regarding respiratory syncytial virus (RSV) burden and prevention.

**Methods:**

An online survey was conducted in the Ipsos Access Panel Online database using two questionnaires, respectively addressed to a large representative sample of the French general population, including at‐risk individuals and independent healthcare professionals. The main topics covered were epidemiology and risk groups, clinical presentations, perceived dangerousness, and attitudes towards preventive measures.

**Results:**

In October 2022, 5000 individuals representing the French general population and 501 independent healthcare professionals, including pharmacists, physicians, and nurses, were surveyed.

Healthcare professionals acknowledge, at 60%, having limited knowledge of respiratory viruses, impacting the care and advice provided to their patients. Both caregivers and patients predominantly call for better education on the subject, leading to a rethink of seasonal awareness campaigns.

More than 90% of the French population and around two‐thirds of healthcare professionals are insufficiently aware of RSV infection.

Vaccination recommendations against respiratory viral infections are widely insufficient in the general population and among vulnerable individuals, despite the majority being willing to do so with detailed information. Eighty‐three percent of at‐risk individuals are inclined to be vaccinated against RSV to protect themselves or others.

**Conclusion:**

The French population and healthcare professionals are insufficiently informed about RSV. Rethinking public health information campaigns will be of paramount importance considering the recently approved RSV vaccines for older adults.

## 1 Introduction

Respiratory viral infections are both common and potentially severe. Their incidence and microbial diversity have risen, with notable changes in the last decade primarily driven by greater awareness and testing capabilities [1, 2, 3, 4]. Pneumonia resulting from these pathogens is more common and severe in the elderly and those with comorbidities, particularly in immunocompromised individuals [5]. Due to the development of new treatments for cancer and autoimmune diseases, the number of patients at high risk of severe infections has increased significantly [6, 7]. This trend highlights the need for global and targeted public health prevention measures, including developing new vaccines and enhancing existing ones. However, even among those most at risk [8], inadequate vaccination coverage is currently a critical issue.

Respiratory syncytial virus (RSV) is an excellent example of a pathogen that is (i) becoming increasingly known of being burdensome on the healthcare system with its high morbidity rate in elderly, immunocompromised patients and those with chronic conditions, (ii) having new preventive tools available. Three vaccines, one monovalent adjuvanted protein vaccine (RSVpre‐F3), one bivalent nonadjuvanted protein vaccine (RSVpre‐F), and one monovalent mRNA vaccine (mRNA‐1345) have been granted marketing authorization by the Food and Drug Administration and European Medical Agency in adults aged 60 years and over in 2023 and 2024. Furthermore, the RSVpre‐F has been approved in pregnant women to protect newborns. These vaccines are recommended for people of different ages and with various risk factors worldwide. In July 2024, the French NITAG (National Immunization Technical Advisory Group) recommended RSV vaccination for those over 75 years, those over 65 with a chronic cardiac or respiratory condition, and pregnant women. Additionally, nirsevimab, a long‐acting monoclonal antibody for RSV, has been available and recommended in France for infants in their first year of life since winter 2023/2024, with the option of maternal immunization left to the parents. Both nirsevimab and maternal RSVpre‐F have received reimbursement approval. RSV vaccines for older adults are still pending reimbursement.

More than ever, we must provide clear and appropriate information to these populations regarding viral respiratory infections and ways to prevent them.

This study aimed to provide an overview of the knowledge of a broad sample of French individuals from the general population and their caregivers concerning RSV infection and prevention.

## 2 Methods

### 2.1 Population

This is a French prospective web‐based survey conducted by Ipsos (a multinational market research firm) online from October 21, 2022, to November 4, 2022, using two validated questionnaires: one for a large representative sample of the French general population, including at‐risk individuals, and the other for healthcare professionals (HCPs).

The two samples were randomly drawn with probability‐based sampling from the Ipsos database of adult eligible panelists. Ipsos developed a loyalty program for respondents, including a reward system based on accumulating points.

In the general population group, at‐risk individuals were identified based on the questions about chronic conditions. This group includes individuals with chronic pathologies such as cardiovascular diseases (congestive heart failure and ischemic heart disease), respiratory diseases (severe asthma and COPD), obesity, diabetes, autoimmune diseases, cancer, chronic renal or hepatic failure, and individuals over the age of 60 years. The HCPs surveyed were liberal French doctors, pharmacists, or nurses.

To ensure the quality of reported data, we monitored and potentially excluded responses that were too fast, overlooked or illogical.

We obtained a representative sample in both groups by carrying out a purposive draw to overrepresent targets with below‐average participation rates.

The data were weighted using a *multilevel calibration* method based on gender, age, and geographic origin, which exactly balances the first‐order margins and *approximately* balances interactions, prioritizing balance in lower‐order interactions over higher‐order interactions.

When sample was sufficient, results were provided for several subgroups within the at‐risk population, including individuals over 60 years of age, patients with cardiovascular or respiratory diseases.

### 2.2 Data Collection

The initial questions were designed to assess the understanding of the general population and HCPs about the epidemiology of viral infections, at‐risk populations, and associated hazards, specifically focusing on RSV respiratory infections. Respondents evaluated their knowledge of respiratory viruses, and health professionals provided information about what they could share with patients regarding these infections. Participants were also asked if they felt sufficiently informed; those who did not were invited to express a desire for more information. Additionally, respondents indicated their willingness to receive vaccinations for various respiratory infections, with a particular emphasis on RSV.

### 2.3 Statistics

Data were collected using the CAWI method (CAWI = computer‐assisted web interviewing).

The reliability of the study's results was evaluated at different stages.

In the pre‐analytical phase, we ensured reliability by using validated standards for the questionnaire. The questionnaire was tested by two different Ipsos evaluators before distribution but no pilot test was performed. During the survey, we maintained reliability by optimizing the randomness of sample selection through a random draw from eligible groups. We also monitored current participation, attrition, and dropout rates. In the post‐analytical phase, the results were presented using 95% confidence intervals (95CI).

### 2.4 Ethical Considerations

Ipsos, as the data controller, guarantees compliance with all relevant privacy, security, and data protection laws and regulations, including the EU Data Protection Regulation 2016/679 (GDPR) and e‐privacy regulations. It identifies the legal grounds for contacting eligible individuals and implements necessary security measures to safeguard their information, including establishing a suitable data retention period. Ipsos provides clear notice to and obtains explicit consent from individuals for using their data in service provision and deliverable creation, including participation in surveys. The final report analyzed and presented only aggregate data, aligning with the international standard ISO 20252 for market, social, and opinion research.

Participants were asked to complete an online informed consent form prior to accessing the questionnaire. Data collection was anonymous, allowing participants to access their responses. The ethics committee IRB of Nîmes University Hospital approved the study (number 22.03.10).

## 3 Results

### 3.1 Population

Overall, 5000 and 501participants were included in the general population and HCP groups, respectively. Participants' characteristics are displayed in Tables 1 and 2.

**TABLE 1:** Characteristics of the “general population” sample (*N* = 5000).

Sex	Female: 2600 (52%) Male: 2400 (48%)
Age	< 35 yo: 1250 (25%) 35–59 yo: 2100 (42%) ≥ 60 yo: 1650 (33%)
Agglomeration category (inhabitants)	< 2000:1150 (23%) 2000–19,999:850 (17%) 20,000–99,999:700 (14%) ≥ 100,000:1500 (30%) Paris: 800 (16%)
Professional activity	Workers: 2800 (56%) Retired: 1400 (28%) Students/unemployed: 800 (16%)
Number of people in the household	1:1100 (22%) 2:1900 (38%) 3 or more: 2000 (40%)
Number of children in the household	None: 3400 (68%) 1:850 (17%) 2 or more: 750 (15%)
Level of education	Before baccalaureate: 1200 (24%) Baccalaureate or greater: 3800 (76%)
Comorbidities	Chronic vascular disease: 800 (16%)^a^ Obesity: 700 (14%) Chronic respiratory disease: 550 (11%)^b^ Diabetes: 450 (9%) Chronic heart disease: 400 (8%)^c^ Auto‐immunity: 400 (6%) Cancer: 250 (5%)^d^ Chronic Kidney Disease: 150 (3%) Chronic liver disease: 100 (2%)

^a^ Including chronic hypertension, thrombosis.

^b^ Including COPD.

^c^ Including cardiac insufficiency, cardiac rhythm disorders, myocardial infarction.

^d^ Within the past 3 years.

**TABLE 2:** Characteristics of the “healthcare professionals” sample (*N* = 501).

	Physicians (*N* = 204)	Pharmacists (*N* = 195)	Nurses (*N* = 102)
Age	< 50yo: 81 (40%) ≥ 50 yo: 123 (60%)	< 50yo: 83 (43%) ≥ 50 yo: 112 (57%)	< 50yo: 74 (73%) ≥ 50 yo: 28 (27%)
Sex	Male: 118 (58%) Female: 86 (42%)	Male: 88 (45%) Female: 107 (55%)	Male: 13 (13%) Female: 89 (87%)
Place of exercise	Individual private practice: 77 (38%) Private practice group: 98 (48%) Health center: 29 (14%)	Private: 195 (100%)	Hospital: 75 (74%) Private: 27 (26%)

The general population sample was slightly representative of the French population [9], with almost the parity between women and men (Table 1). The majority lived in urban areas and were current workers, with a level of education at least equivalent to the baccalaureate. One third of the respondents were over 60 years and were part of the 1981 individuals (39.6%) identified as being at risk of severe infections due to age or chronic conditions, primarily chronic cardiovascular disease, obesity, and chronic respiratory diseases within the sample.

The HCP sample included 204 physicians, 195 pharmacists and 102 registered nurses (Table 2). Most of them were independent workers, except for nurses. Their characteristics were broadly similar to those of the French caregiver's population [10].

For example, the majority of physicians doctors were male (63%) and over 50 years old (60%), while the majority of nurses were female (85%) and under 50 years old (72%).

### 3.2 Current Knowledge About Viral Respiratory Infections

Forty‐six percent of the general population and 60% of the HCPs considered themselves well‐informed on respiratory infections.

Awareness was low among those most at risk of developing severe forms of the disease, with only 58% aware that they were more vulnerable than the general population. Older people did not consider themselves more at risk than their younger counterparts. Among those, at‐risk people with chronic respiratory disease and autoimmune diseases appeared to be the most educated.

This translates into risky health behaviors, with less than two‐thirds of those at risk and experiencing respiratory symptoms seeking medical attention in time.

Participants from the general population had limited knowledge of RSV, with 94% either needing to be made aware of what it is or claiming to have more accurate knowledge. More than half believed that RSV is harmless in adults. Additionally, 46% did not know or deny that it is a pathogen that causes lower respiratory infections. Furthermore, 68% of those most at risk of severe forms of this virus were not aware or unsure that it could be fatal to them.

Caregivers also had a high level of ignorance about this infection. One‐quarter of physicians were unaware that RSV can be dangerous for adults, and 40% considered it less severe than influenza.

However, almost half of the surveyed physicians reported having hospitalized at least one patient for RSV infection during the year. More than 50% of them acknowledged that RSV can have severe consequences on their patients' autonomy, leading to significant middle‐term outcomes. This underscores the need for preventive measures and increased awareness about RSV.

There is encouraging clarity about this lack of knowledge and a willingness to address it, with more than 90% of HCPs and 70% of the general population claiming for more information about respiratory viruses and their potential dangerousness.

### 3.3 Public Information About Respiratory Infections From Healthcare Professionals

Vaccination recommendations for influenza, SARS‐CoV‐2, and pneumococcal diseases were low, with only 59% of HCPs encouraging most of their patients to be vaccinated. Thirty‐four percent gave vaccination recommendations only to their patients at risk of severe disease.

There was also a large gap between the information HCPs believed they provided and the information received by their patients. For example, only 23% of at‐risk patients appeared to have been recommended pneumococcal vaccine, 45% for influenza, and 64% for SARS‐CoV2.

There was a difference in the perception of this need for more communication between general practitioners and hospital staff. For example, 50% of independent nurses felt that their patients were not well informed on the subject, a feeling shared by 34% of their hospital counterparts. Eighty‐two percent of primary care physicians felt there was a lack of vaccine information, while only 56% of hospital physicians had this opinion. However, there was no significant difference in awareness of this lack of information between GPs in individual or group practices.

Most respondents from the general population considered their general practitioners and specialists to be reliable sources of information about vaccinations. They typically turn to their doctor first when experiencing symptoms suggestive of a respiratory infection, with 73% of the public reaching out to a physician in these cases. However, recommendations for individuals at risk were insufficient. Only 49% of those at risk were informed by their physician that an acute respiratory infection could pose a greater danger to them, and just 71% were advised to get vaccinated against some of these diseases.

### 3.4 Behaviors About Preventive Measures, Opinions About Vaccines

In addition to this lack of information, health professionals sometimes faced strong vaccine hesitancy, reported in 12%, 23%, and 40% of cases for influenza, SARS‐CoV2, and pneumococcal vaccines, respectively. For RSV, caregivers and patients are inclined to add a targeted vaccine to the arsenal of preventive measures against seasonal infections. Eighty‐nine percent of caregivers would recommend it, but only 29% would surely do so. Eighty percent would consider recommending it to the elderly, regardless of their medical history, and only 62% would recommend it to parents of young children.

Sixty‐four percent of the general public would be willing to be vaccinated if it prevented severe forms of the disease, but only 23% would definitely do so. However, there is a strong support among the frail, with 83% expressing willingness when it is explained that the vaccine will protect them or those around them against circulation and severe forms of the virus.

## 4 Discussion

Our study had the advantage of surveying a large representative sample of French people, isolating an at‐risk population, and examining the knowledge of HCPs.

On the eve of the large‐scale commercialization of vaccines against RSV, the issue of vaccine acceptance is more relevant than ever.

Our findings among the French population are the same as in other Western countries: Public knowledge of the epidemiology and potential seriousness of viral respiratory infections is mainly inadequate, given their frequency and impact on public health and the healthcare system [11]. This is especially true for the RSV. Indeed, we found that 94% of the general population participants claimed more information about RSV, and 68% of those most at risk of severe forms were unaware of its potential lethality, highlighting the crucial need for education and awareness campaigns.

A similar survey was conducted in the United States in May and June 2022 among people aged over 60 or under 60 year but with diabetes or chronic pulmonary or cardiovascular disease [12]. More than 55% had never heard of RSV, especially in the older age groups, and 19% did not know that it can cause severe forms of the disease.

In China in 2023, Wang et al. [13] sent a questionnaire to 2133 participants representing the general population to assess their knowledge of RSV. The results showed that 24.3% of respondents had never heard of this, but the majority were interested in learning more, and 68.4% were willing to be vaccinated against the virus. Factors that positively correlated with vaccine acceptance were age, level of education and standard of living, medical history, and knowledge of RSV.

The need for more education at all system levels explains why healthcare providers feel powerless to educate their patients about appropriate health behaviors properly. Our study reminds us of the vital role that physicians and paramedics play in health education despite their lack of knowledge on the subject, knowing that most of those surveyed expressed a willingness to receive the RSV vaccine if it reduces transmission and the risk of severe forms.

RSV vaccine hesitancy, as for all vaccines, might be an issue shortly. The 3C model of vaccine hesitancy considers complacency, confidence, and convenience as contributing factors. Complacency occurs when perceived risks of vaccine‐preventable diseases are low, and vaccination is not deemed a necessary preventive action. For RSV, historical literature and recent media reports mainly focus on its incidence among children and the resulting burden on primary care and hospital services. It is thus unsurprising that both clinicians and the public view RSV as exclusively a childhood concern.

Confidence, a key component of the 3C model, is built on trust in the effectiveness and safety of vaccines. This trust is fostered by the rigorous testing and monitoring processes that vaccines undergo before they are approved for use which has been the case for the three authorized RSV vaccines. The duration of time on the market may also affect confidence, as it enables the collection of real‐world data confirming efficacy and safety.

Convenience, the third component of the 3C model of vaccine hesitancy, plays a significant role in vaccine uptake. Factors such as physical availability, affordability, geographical accessibility, and the appeal of immunization services can all positively influence vaccination rates. For RSV, the vaccine's duration of protection across at least two seasons and the option of coadministration are expected to further enhance the convenience of being vaccinated.

It is essential that public health campaigns are designed to target not only the general population but also HCPs. This targeted approach can help bridge the gap in healthcare education and reduce vaccination hesitancy. It is crucial to understand the rationale behind health behaviors to fully appreciate their benefits. This understanding is often lacking, leading to inadequate education and a reluctance to vaccinate. The public often fills these gaps in understanding through less reliable sources, such as the Internet and social networks, which can negatively impact vaccine opinion [14, 15, 16, 17].

Therefore, Berrada et al. defined three main themes in their qualitative study concerning vaccination hesitancy: restoration of trust in vaccine policy, improvement of the initial and further training of healthcare workers, and better communication with the population [18].

Our study has several limitations that we must acknowledge. First, the design of the study may have introduced social desirability bias, leading to an overestimation of the frequency of information and positive attitudes towards vaccination among HCPs and the general population. Second, the study was conducted during the COVID‐19 pandemic, which could have influenced respondents' knowledge about respiratory infections and their opinions on vaccination. Lastly, our sample from the general population included only 40% of at‐risk patients, whose responses are particularly significant as they are the primary targets of vaccine recommendations.

In conclusion, we found that French people and their caregivers have limited knowledge about respiratory viruses including RSV and often underestimate its dangerousness, even those who are at risk of developing severe forms. Given the prevalence and morbidity of these diseases large‐scale awareness campaigns must be conducted primarily among HCPs, who remain one of the most important sources of health information for the public. These issues are more relevant than ever on the eve of the large‐scale use of vaccines against RSV.

## Role of the Funding Source

Ipsos was funded by Janssen.

Janssen had no role in data analysis, decision to publish or preparation of the manuscript.

## Author Contributions

**Joshua Puel:** writing – original draft. **Katia Sosnowiez:** methodology, conceptualization. **Robin Stephan:** writing – review and editing. **Albert Sotto:** writing – review and editing. **Paul Loubet:** conceptualization, supervision, writing – original draft, writing – review and editing, validation, methodology.

## Conflicts of Interest

P.L. has received payment or honoraria for lectures, presentations, speakers bureau, manuscript writing, or educational events from AstraZeneca, GlaxoSmithKline, Janssen, Moderna, Merck Sharp & Dohme, Pfizer, Sanofi Pasteur, and Seqirus. K.S. is an employee of Janssen and may hold shares or stock options in the company.

The other authors have no competing interest.

### Peer Review

The peer review history for this article is available at https://www.webofscience.com/api/gateway/wos/peer‐review/10.1111/irv.70103.

## Data Availability

The authors confirm that the data supporting the findings of this study are available within the article. Raw data supporting this study's findings are available from the corresponding author upon reasonable request.
